# Evaluation of hypointense liver lesions during hepatobiliary phase MR imaging in normal and cirrhotic livers: is increasing flip angle reliable?

**DOI:** 10.1038/srep18942

**Published:** 2016-01-06

**Authors:** Yu-dong Xiao, Cong Ma, Jun Liu, Hua-bing Li, Zi-shu Zhang, Shun-ke Zhou

**Affiliations:** 1 Department of Radiology, the Second Xiangya Hospital of Central South University, Changsha, China

## Abstract

Gd-EOB-DTPA is a newly developed liver specific magnetic resonance contrast agent, which is widely used for focal liver lesion (FLL) detection and liver function evaluation. However, it has been demonstrated that hepatocytes uptake of Gd-EOB-DTPA obviously decreased in cirrhotic liver, and cirrhotic liver parenchyma may show reduced enhancement in hepatobiliary phase, which would result in decreased liver-to-lesion contrast (LLC) and liver to lesion signal intensity ratio (LLSIR). Therefore, it is important to improve the image quality in cirrhotic liver, as it may alter therapeutic strategy. In this paper, we have shown adjustments of the flip angle (FA) provides a simple step to achieve better image quality for evaluation of FLLs, especially to those patients with severe liver cirrhosis. On the basis of our quantitative analysis, both of the LLC and the LLSIR with high FA protocol were always higher than those of low FA protocol. Additionally, on high FA images, more FLLs were detected, peritumoral invasion was found, boundary of the tumor was more remarkably, and better visualization of bile duct was observed. In conclusion, for the patient with severe liver cirrhosis, increasing FA can obviously improve the image quality, which is helpful for FLLs depiction.

Gadolinium ethoxybenzyl diethylenetriamine pentaacetic acid (Gd-EOB-DTPA, Primovist, Bayer Schering Pharma, Berlin, Germany) is a newly developed liver specific magnetic resonance (MR) contrast agent which is widely used for focal liver lesion (FLL) detection and liver function evaluation^1^,^2^,^3^,^4^. This contrast agent differs from conventional Gd-based extracellular contrast agent because it not only can be used for dynamic phase imaging, but are also taken up by hepatocytes and excreted into bile^5^. The hepatocyte-specific uptake of Gd-EOB-DTPA is thought to be mediated by an active membrane transport system such as organic anion-transporting polypeptide (OATP), and it has been demonstrated that hepatocytes uptake of Gd-EOB-DTPA obviously decreased in cirrhotic liver^6^. Cirrhotic liver parenchyma may show reduced enhancement in hepatobiliary phase (HBP), which would result in decreased liver-to-lesion contrast^7^,^8^,^9^. Therefore, it is important to improve the image quality in cirrhotic liver, as it may alter therapeutic strategy.

Flip angle (FA) is an important MR scan parameter, which has a certain effect on the image quality^10^. Previous studies often use a low FA, ranged from 9° to 15°, to obtain both contrast-enhanced dynamic images and HBP images with Gd-EOB-DTPA^1^,^2^,^3^,^4^,^7^,^8^,^9^. However, although it seems to be suitable employing such a low FA for dynamic perfusion phase imaging with extracellular contrast agents like Gd-DTPA, it may not yield the optimal option during the HBP phase of Gd-EOB-DTPA-enhanced MR examination. A cohort study conducted by Haradome H *et al.*^11^ reveals that a high FA could significantly improve the detection rate of FLLs. To the best of our knowledge, there is no published research using different FA to compare the image quality of Gd-EOB-DTPA-enhanced MR in normal and cirrhotic livers.

This paper, therefore, aims to compare the image quality of Gd-EOB-DTPA-enhanced MR between conventional low FA and high FA in normal and cirrhotic livers.

## Results

### Quantitative analysis

Signal to noise ratio (SNR) of the lesion and the liver parenchyma was calculated. Mean SNR_lesion_ and SNR_liver_ with low FA and high FA protocol in each phase are summarized in Table 1. At the HBP of 5 min, 10 min, 15 min, 20 min, the SNR_lesion_ with low FA was significantly higher than that of SNR_lesion_ with high FA, while the SNR_liver_ with low FA was significantly lower than that SNR_liver_ with high FA. However, at the unenhanced phase, the SNR_lesion_ and SNR_liver_ with low FA were similar to those of SNR with high FA.

**Figure 1:**
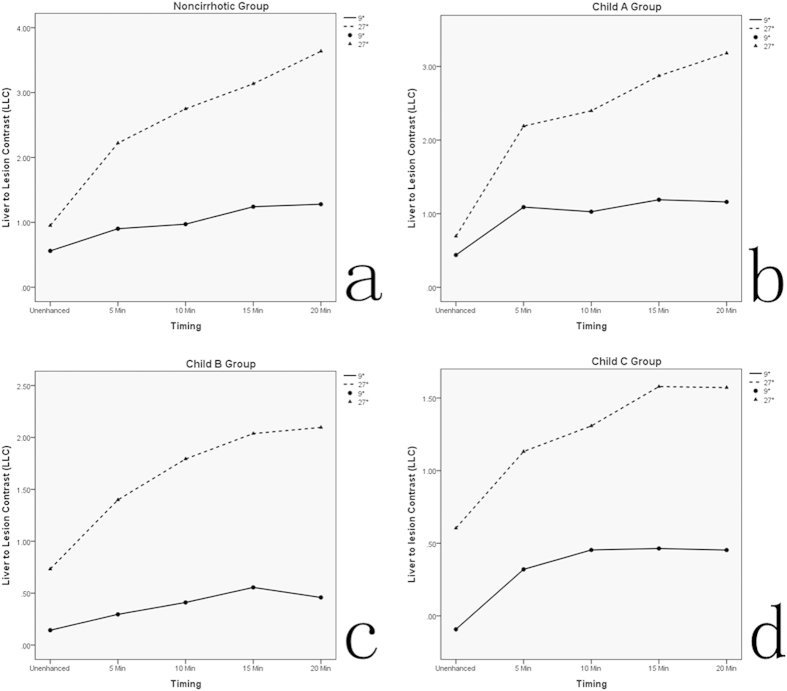
The graph shows the trend of liver to lesion contrast (LLC) change with high flip angle (FA) and conventional low FA protocol over the time course before and after contrast agent injection in each group.

The liver to lesion contrast (LLC) with high FA was always higher than that of low FA (P < 0.05). The LLC with both low FA and high FA protocol in all groups showed a stepwise increase over time from unenhanced phase to 15 min after Gd-EOB-DTPA administration. Nevertheless, in Child B group and Child C group, the LLC with low FA was obviously reduced since the timing of 15 min to 20 min. However, compared to low FA protocol, the LLC with high FA in Child B and Child C group tended to flatten since the timing of 15 min to 20 min. Figure 1 summarizes the tendency of LLC change over the time course before and after contrast agent injection in each group.

**Figure 2:**
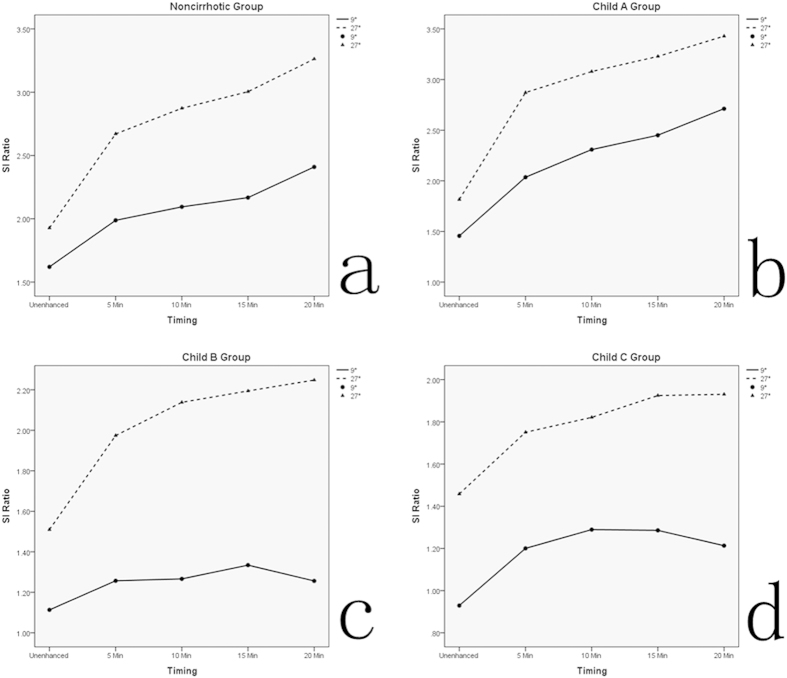
The graph shows the trend of corresponding liver to lesion signal intensity ratio (LLSIR) change with high flip angle (FA) and conventional low FA protocol over the time course before and after contrast agent injection in each group.

The liver to lesion signal intensity ratio (LLSIR) with high FA was always higher than that with low FA in all groups (P < 0.05). The corresponding LLSIR with low FA and high FA protocol in each group was summarized in Fig. 2. In noncirrhotic group and Child A group, the LLSIR with both low FA and high FA protocol was gradually increased over time from unenhanced phase to HBP (20 min). In Child B group and Child C group, the LLSIR with low FA was significantly reduced since the timing of 15 min to 20 min. However, the LLSIR with high FA was obviously different to that of low FA since the timing of 15 min to 20 min. In Child B group, LLSIR with high FA was sustained increased from the timing of 15 min to 20 min, and in Child C group, the LLSIR with high FA tended to flatten from the timing of 15 min to 20 min.

Additionally, the mean LLC and the mean LLSIR in all groups between high FA and low FA protocol at unenhanced phase, 5 min, 10 min, 15 min, and 20 min after contrast agent administration were summarized in Table 2.

### Qualitative analysis

**Figure 3:**
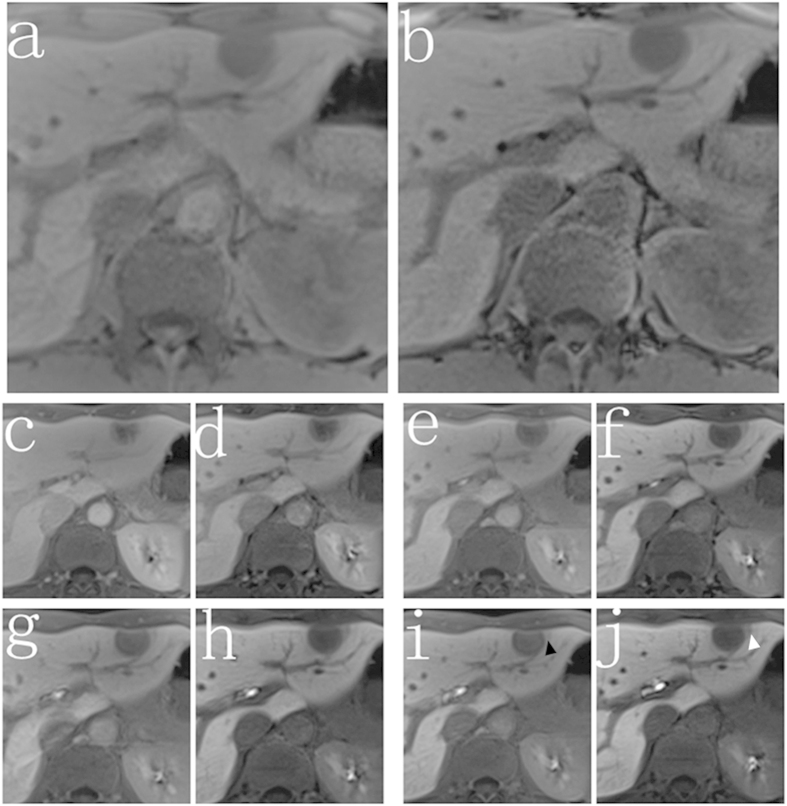
Images from a 37-year-old man with hepatic metastasis of colorectal cancer (noncirrhotic group). The 3D T1-weighted VIBE sequence with either conventional low FA protocol (**a,c,e,g,i**) or high FA protocol (**b,d,f,h,j**) were acquired at the unenhanced phase (**a,b**) and 5 min (**c,d**), 10 min (**e,f**), 15 min (**g,h**), 20 min (**i,j**) after contrast agent injection. Peritumoral invasion was shown more clearly at the high FA image (white arrow) than the low FA image (black arrow).

**Figure 4:**
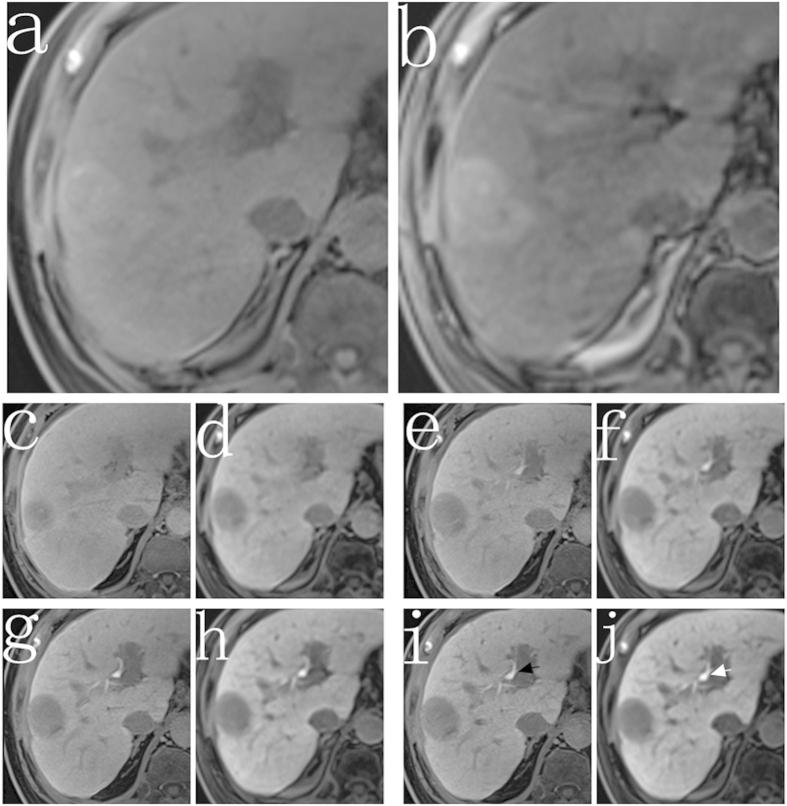
Images from a 78-year-old man with viral hepatitis C-induced cirrhosis (Child A group). The 3D T1-weighted VIBE sequence with either conventional low FA protocol (**a,c,e,g,i**) or high FA protocol (**b,d,f,h,j**) were acquired at the unenhanced phase (**a,b**) and 5 min (**c,d**), 10 min (**e,f**), 15 min (**g,h**), 20 min (**i,j**) after contrast agent injection. The SI of the bile duct was significantly higher at high FA image (white arrow) than that of low FA image (black arrow).

**Figure 5:**
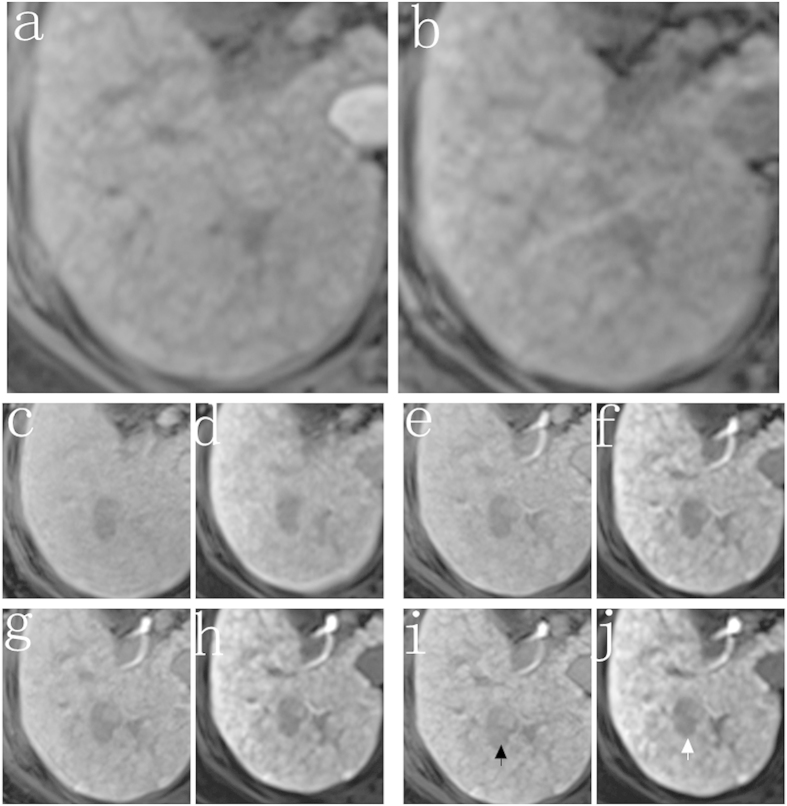
Images from a 61-year-old woman with viral hepatitis B-induced cirrhosis (Child B group). The 3D T1-weighted VIBE sequence with either conventional low FA protocol (**a,c,e,g,i**) or high FA protocol (**b,d,f,h,j**) were acquired at the unenhanced phase (**a,b**) and 5 min (**c,d**), 10 min (**e,f**), 15 min (**g,h**), 20 min (**i,j**) after contrast agent injection. The boundary of the tumor was more clearly at high FA image (white arrow) than the low FA image (black arrow).

**Figure 6:**
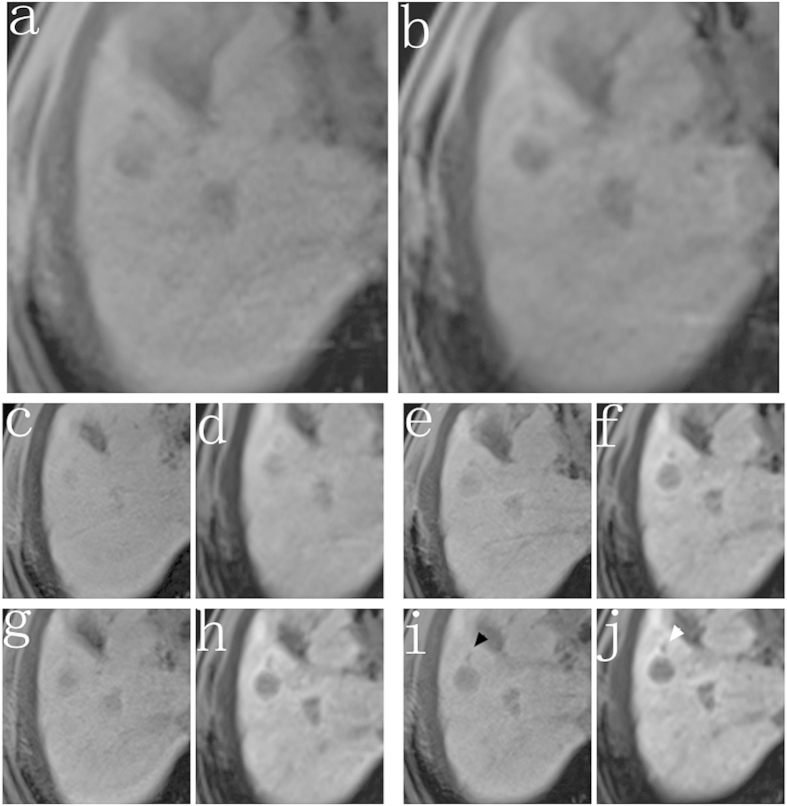
Images from a 52-year-old man with viral hepatitis B-induced cirrhosis (Child C group). The 3D T1-weighted VIBE sequence with either conventional low FA protocol (**a,c,e,g,i**) or high FA protocol (**b,d,f,h,j**) were acquired at the unenhanced phase (**a,b**) and 5 min (**c,d**), 10 min (**e,f**), 15 min (**g,h**), 20 min (**i,j**) after contrast agent injection. A satellite foci was detected at high FA image (white arrow).

In all groups, imaging details were more clearly depicted on high FA images. Peritumoral invasion was found in more patients on high FA image (n = 24) compared to low FA image (n = 15) (P = 0.041) (Fig. 3). The SNR of bile duct was significantly higher at high FA protocol than low FA protocol (274.3 ± 21.6 vs 168.6 ± 23.7, P = 0.024) (Fig. 4). Boundary of the tumor was more remarkably (Fig. 5). More FLLs were detected at high FA protocol (n = 174) in comparison with low FA protocol (n = 152) (P = 0.046) (Fig. 6).

### Inter-observer agreement between two reviewers

Based on the intraclass correlation coefficient (ICC) analysis, there was satisfactory correlation between two reviewers for signal intensity (SI) calculation with the ICC value of 0.84, which indicated acceptable inter-observer agreement.

## Discussion

Gd-EOB-DTPA, a liver specific MR contrast agent, is gradually taken up by functional hepatocytes and excreted via the biliary pathway^12^. The hepatocytes uptake and excretion of this contrast agent is mediated by the active membrane transport system such as OATP, especially OATP1B3, and the multidrug resistance-associated protein (MRP), especially MRP2^13^. However, it has been described that hepatocytes uptake of Gd-EOB-DTPA is reduced owing to the decreased expression of transporting proteins in advanced liver cirrhosis, which cause a poor LLC and LLSIR^14^. A poor LLC and LLSIR may make it difficult to depict small FLLs, which may have a potential impact on clinical decision making. Besides, Golfieri R, *et al.*^15^ reveals that the detection of FLLs in patients with cirrhosis is reduced on Gd-EOB-DTPA-enhanced MR scans. Therefore, it is of great value to investigate the optimal parameter for MR detection of FLLs, especially in patients with severe liver cirrhosis. In the present study, we have shown that adjustment of the FA provides a simple solution to achieve better image quality in evaluation of FLLs.

FA is an important MR scan parameter, which has an certain effect on image quality. Almost all previously published studies used a standard low FA, ranged from 9° to 15°, to obtain both contrast-enhanced dynamic images and HBP images with Gd-EOB-DTPA^7^,^8^,^9^. However, it seems to be inadequate to employ such a low FA during HBP phase of Gd-EOB-DTPA-enhanced MR, because the longitudinal magnetization in gradient echo sequence imaging with short repetition time is incompletely recovered^16^. It has been reported that the estimated optimal FA for tissues with long T1 relaxation times is lower such as unenhanced lesions, while that of tissues with short T1 relaxation times is higher such as liver parenchyma^17^. Additionally, this change can also suppress the signal intensity of nonhepatocyte-containing tissues such as skeletal muscle^18^. Recently, a cohort study conducted by Bashir MR *et al.*^19^ has proved that a high FA, ranged from 25° to 40°, can significantly increase the signal intensity of liver parenchyma and biliary system. Frydrychowicz A *et al.*^20^ suggested that the optimal FA for liver lesion detection was 25°–30°, and for assessment of biliary system was 45°. Thus, a high FA of 27° was used in our study to perform Gd-EOB-DTPA-enhanced MR scans. Quantitative and qualitative comparison between Gd-EOB-DTPA-enhanced MR with low FA and that of with high FA was also performed.

On the basis of our quantitative analysis, the present study indicates that both the LLC and the LLSIR with high FA protocol are always higher than those with low FA protocol. The result is that the overall image quality at high FA protocol are improved comparing with those of low FA. This improved visualization of the FLLs at high FA protocol may be attributed to the greater difference in signal intensity between FLLs and surrounding tissue. In addition, in noncirrhotic group and Child A group, the LLC and the LLSIR are gradually increased with either high FA or low FA protocol. However, in Child B group and Child C group, the LLC and the LLSIR are significantly reduced at low FA protocol from the timing of 15 min to 20min after contrast agent administration, which is different to that of high FA protocol. Therefore, in case of patients with severe liver cirrhosis such as Child B or C, increasing the FA during HBP MR imaging can improve the image quality, which provides more detailed imaging information.

In the qualitative analysis of each group, details are more clearly depicted on high FA protocol images. More FLLs and more peritumoral invasion were detected on high FA images. Better visualization of bile duct was observed. Boundary of the tumor was more remarkably. As we all know, detailed image description is important for patient’s clinical decision making, especially for those patients with Child class B or C. For instance, according to the Barcelona Clinic Liver Cancer (BCLC) staging system^21^, patients with single nodule may choose surgical therapy, but for the patients with multiple lesions, they may choose transarterial chemoembolization (TACE) as the treatment option.

However, one important consideration in increasing the FA in any pulse sequence is that of a field strength-independent nine-fold increase in specific absorption rate (SAR)^22^,^23^. This is a particular challenge when using 3 Tesla MR scanner. Previous studies have reported that in order to maintain a higher FA at 3 Tesla MRI, increasing the slice thickness, increasing the radiofrequency pulse duration, decreasing the number of phase encoding steps, and decreasing the number of images acquired may be effectively to manage the SAR^24^. In the present study, all MR scans were performed under the SAR limits.

There are several limitations to our study. First, the number of patients enrolled was relatively small. Second, not all the FLLs were histopathologically confirmed, as the histopathology was the gold standard for the diagnosis of liver lesions. In the present study, only 58 liver lesions were confirmed by histopathological examination, which might have a potential impact on the interpretation of results to the study. Finally, only the high FA of 27° and the conventional 9° FA were compared, which was too limited to assess the FLLs. Multi-FAs should be considered in the futher studies.

In conclusion, we found significantly higher LLC and LLSIR on Gd-EOB-DTPA-enhanced MR images using a high FA protocol in comparison with the conventional low FA protocol. In those patients with Child B or C class, increasing the FA can obviously improve the image quality, which is helpful for liver lesion depiction. Thus, we highly recommend a higher FA protocol to perform Gd-EOB-DTPA-enhanced MR, especially in those patients with severe liver cirrhosis.

## Methods

### Patients

Institutional review board approval in accordance with the approved guidlines from our hospital was obtained for this retrospective study and written informed consent was obtained from all patients.

From September 2013 to December 2014, a total of 136 consecutive patients who having or with suspected FLLs underwent Gd-EOB-DTPA-enhanced MR of the liver were enrolled in this retrospective study. Of the 136 patients, 22 patients were excluded based on the following criteria: incomplete full MR examination (n = 13); poor imaging quality (n = 6); previously received radiofrequency ablation therapy or Argon-Helium cryoablation (n = 3). Finally, the study population comprised these 114 patients (71 men, 43 women) ranged in age from 26 to 81 years with a mean age of 54.7 ± 10.8 years.

Of the 114 patients, 39 patients were in noncirrhotic group, 36 patients were in Child A group, 23 patients were in Child B group, and 16 patients were in Child C group. The causes of cirrhosis included type B hepatitis (n = 53), type C hepatitis (n = 11), alcohol abuse (n = 8), primary biliary cirrhosis (n = 2), autoimmune hepatitis (n = 1). The diagnosis of liver cirrhosis was confirmed by histology in 53 patients (surgical resection, n = 36; biopsy, n = 17) or was made based on combination of physical findings, biochemical tests, and radiological imaging features in 22 patients. Detailed information was listed in Table 3.

### Standard of reference

**Figure 7:**
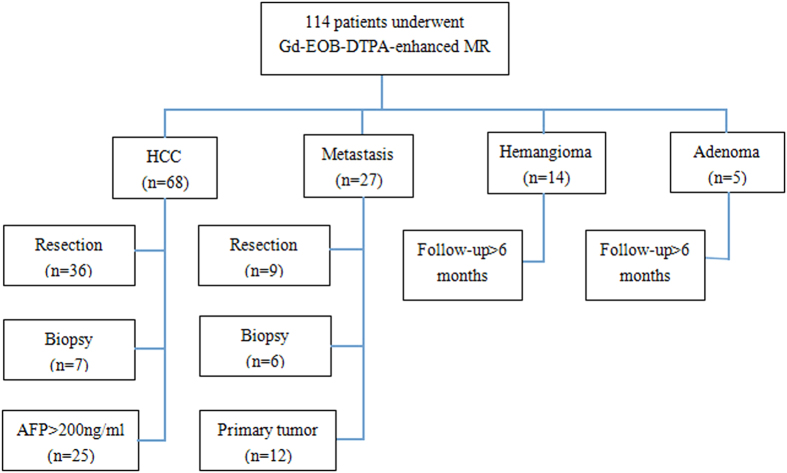
Flowchart of patients and lesions. One hundred and fourteen consecutive patients who underwent Gd-EOB-DTPA-enhanced MR were enrolled in the study.

Final diagnosis of FLLs was confirmed by either surgical histology (hepatocellular carcinoma (HCC), n = 36; metastasis, n = 9) or biopsy (HCC, n = 7; metastasis, n = 6) or elevating tumor marker over 2 months (α-fetoprotein (AFP) > 200ng/ml, HCC, n = 25) or surveillance cross-sectional imaging with known primary tumor (metastasis, n = 12) or surveillance cross-sectional imaging without change in size over 6 months (hemangioma, n = 14; adenoma, n = 5). Moreover, in case of the multiple lesions, only the largest lesion had been used for analysis. Detailed information was shown in Fig. 7.

### MR imaging technique

MR imaging was performed using a clinical whole body 3 Tesla system (Magnetom Skyra, Siemens Healthcare, Germany) with a combination of body and spine array coil elements (18-channel body matrix coil, 24-channel spine matrix coil). A T1-weighted volume interpolated breath hold examination (VIBE) sequence with fat suppression was acquired before and after the administration of Gd-EOB-DTPA. All images were obtained in the transverse plane covering the entire liver. Gd-EOB-DTPA-enhanced MR protocols were as follows: 64 slices; 3mm section thickness; reconstructed voxel size, 1.3mm × 1.3mm × 3.0mm; measured voxel size, 1.7mm × 1.3mm × 4.5mm; repetition time (TR), 5.67ms; echo time (TE), 1.43ms, field of view, 268 × 330mm. Gd-EOB-DTPA was used at a dose of 0.025mmol/kg body weight and at an injection rate of 2ml/s by 20ml saline flush using a cubital intravenous line. After administration of Gd-EOB-DTPA, the contrast-enhanced images were obtained at the arterial phase (18s), portal venous phase (90s), delayed phase (3min), HP (5min, 10 min, 15 min, 20 min). All images were acquired with both conventional low FA (9°) and high FA (27°) protocols at all time points in the HP, and other parameters were kept constant.

### Imaging analysis

Two radiologists with 25 years and 10 years abdominal imaging experience randomly evaluated all the conventional low FA (9°) and high FA (27°) images that acquired at the unenhanced phase, and the HBP of 5 min, 10 min, 15 min, 20 min. Both radiologists were blinded to the clinical data and to the FA that were used. For quantitative analysis, signal intensity (SI) of liver parenchyma (SI_liver_), FLLs (SI_lesion_), background noise (SI_air_), and paravertebral muscle (SI_muscle_) were measured using regions of interest (ROIs) at the same slice in each sequence. The ROIs were circular or oval in shape, chosen as large as possible (size of ROI ranged from 0.8 cm^2^ to 5.0 cm^2^), avoiding visible vessels and imaging artifacts. In case of multiple lesions, only the largest lesion had been measured.

Signal to noise ratio of the liver (SNR_liver_) is calculated as: SI_liver_/SI_air_. SNR of the lesion (SNR_lesion_) is calculated as SI_lesion_/SI_air._ The liver to lesion contrast (LLC) is calculated as: (SI_liver_ – SI_lesion_)/SI_muscle_. The liver to lesion SI ratio (LLSIR) in each phase is calculated as: SI_liver_/SI_lesion_.

### Statistical analysis

The data was presented as mean ± SD and analyzed using SPSS 19.0 software (SPSS Inc., Chicago, Illinois, USA). The non-parametric Mann-Whitney U Test and Wilcoxon Test were used to compare groups. The intraclass correlation coefficient (ICC) was used to evaluate the inter-observer agreement between two reviewers. Agreement was classified as poor (ICC, 0–0.40), fair to good (ICC, 0.40–0.75), or excellent (ICC, > 0.75). All reported P values were two-sided, and a P value less than 0.05 was considered statistically significant.

## Additional Information

**How to cite this article**: Xiao, Y.-D. *et al.* Evaluation of hypointense liver lesions during hepatobiliary phase MR imaging in normal and cirrhotic livers: is increasing flip angle reliable? *Sci. Rep.* **6**, 18942; doi: 10.1038/srep18942 (2016).

## Figures and Tables

**Table 1:** Mean SNR of lesion and liver with 9° and 27° protocol in each phase.

	Lesions		P	Liver parenchyma		P
	9°	27°		9°	27°	
Unenhanced	24.09	20.79	0.077	44.78	42.09	0.081
5 min	59.89	31.53	0.038	72.92	103.61	0.025
10 min	62.50	31.57	0.022	81.43	117.64	0.019
15 min	60.69	31.46	0.024	85.06	121.78	0.021
20 min	61.33	31.32	0.019	84.64	124.13	0.017

**Table 2:** The mean LLC and mean LLSIR between high FA and low FA.

	LLC			LLSIR		P
	9°	27°	P	9°	27°	
Unenhanced phase	0.39 ± 0.36	0.79 ± 0.52	0.041	1.41 ± 0.45	1.77 ± 0.58	0.057
5 min	0.81 ± 0.64	1.97 ± 1.07	0.025	1.81 ± 0.75	2.54 ± 1.56	0.036
10 min	0.85 ± 0.56	2.33 ± 1.16	0.019	1.89 ± 0.55	2.74 ± 1.21	0.033
15 min	1.03 ± 0.66	2.72 ± 1.22	0.017	1.97 ± 0.78	2.88 ± 1.31	0.029
20 min	1.02 ± 0.64	2.99 ± 1.44	0.013	2.05 ± 0.64	3.01 ± 1.37	0.023

**Table 3:** Clinical information of the 114 patients#.

Parameters	
Age range (mean age)	26–81years (54.7 ± 10.8years)
Gender (n = 114)	Male (n = 71)
	Females (n = 43)
Diagnosis of FLLs (n = 114)	HCC (n = 68)
	Metastasis (n = 27)
	Hemangioma (n = 14)
	Adenoma (n = 5)
Cause of liver cirrhosis (n = 75)	Type B hepatitis (n = 53)
	Type C hepatitis (n = 11) Alchol abuse (n = 8)
	Primary biliary cirrhosis (n = 2)
	Autoimmune hepatitis (n = 1)
Non-cirrhotic liver (n = 39)	Normal liver (n = 39)
Cirrhotic liver (n = 75)	Child A (n = 36)
	Child B (n = 23)
	Child C (n = 16)

^#^In case of the multiple lesions, only the largest lesion had been measured.
